# Progress in Bacteriology

**Published:** 1902-03-01

**Authors:** 


### PROGRESS IN BACTERIOLOGY.

**Diphtheria.**—In a brief communication on the subject of Neisser's test for diphtheria bacilli, Cobbett[1] gives a new method of distinguishing between the true Klebs-Löffler bacillus and the Hofmann variety. Neisser's stain, he finds, fails to show polar bodies in a small proportion of true diphtheria bacilli, and may occasionally show polar bodies in the Hofmann species. Reliance cannot be placed upon the length of the bacilli, for long or giant forms of the Hofmann bacillus are not infrequent. The following procedure is suggested: The film taken from the culture is stained in watery methylene blue (1 in 5). If suspicious forms are present the cover-glass is taken up again, cleansed from the cedar oil, by means of blotting-paper soaked in xylol, and washed for a minute in 5 per cent. acetic acid, and then stained by the ordinary Neisser's method. If, as is frequently the case, it is wished to test one particular group of diphtheria bacilli without removing the slide from the microscope, a drop of 5 per cent. acetic acid is applied to one edge of the cover-slip and the fluid drawn underneath the glass by means of filter-paper. True diphtheria bacilli so treated are partially decolorised, the body being a pale blue and the polar bodies standing out as deep blue masses. This change is instantaneous in the case of the diphtheria bacilli, whilst Hofmann's bacilli do not decolorise so rapidly, and usually show masses of stain remaining in the middle of each half. This acetic acid method is in Cobbett's opinion superior to Neisser's test. Another distinctive staining reaction has been brought forward by Pitfield.[2] He uses three solutions: (1) Silver nitrate, 5 grammes; distilled water, 5c.c.; saturated alcoholic solution of fuchsin, 3c.c. (2) Pyrogallic acid, 1 gramme; sodium hydrate (10 per cent. solution), 5c.c.; distilled water, 10c.c. (3) Carbol fuchsin solution, 10 drops; distilled water, 10c.c. The film is first covered with solution 1, heated until it boils, and kept thus for one minute and then washed. It is then similarly treated with solution 2, and, finally, solution 3 is applied for two minutes, and the cover-glass washed and dried. The body of the diphtheria bacillus is stained a faint pink, and the polar bodies appear as shining black dots. The staining reaction appears to vary according to the virulence of the variety of bacillus. A very careful research into the nature of the diphtheroid forms found in cases of post scarlatinal diphtheria has been made by Williams.[3] For cultural purposes he employed Loeffler's blood serum, and although Neisser's stain was employed, he places more reliance on methylene blue. Gram's stain, although it destroys the beading, is a useful differential stain. Eight faucial, nine nose, and two ear cases, showed typical diphtheria bacilli. In 54 cases atypical bacilli were found, and the nature of these organisms was in 21 cases tested by inoculation experiments. Five proved to be virulent Klebs-Loffler bacilli, five belonged to the Hofmann variety, and the remainder were regarded as modified non-virulent forms of diphtheria bacilli. These results accentuate the necessity of isolating and treating all persons in whom the short atypical varieties have been shown to be present. Lesieur[4] believes that the so-called pseudo-diphtheria bacilli found in healthy persons or in the fauces of persons recently convalescent from diphtheria are true diphtheria bacilli attenuated by the germicidal action of the body secretions. Of 14 persons who had recently been in contact with diphtheria, four had diphtheria bacilli in the nose, six had pseudo-diphtheria bacilli in the nose, and one pseudo-bacilli in both nose and throat. Pseudo-diphtheria bacilli were also present in the noses of seven out of sixteen persons who had not been exposed to infection. In cases of true diphtheria the bacilli taken from the nose become non-virulent much quicker than do those taken from the throat. Gordon Pugh [5] classifies the organisms found into diphtheria as follows:—(1) Virulent diphtheria bacilli, (2) non-virulent diphtheria bacilli, (3) Hofmann's bacilli (pseudo-diphtheria). It is impossible by microscopical examination alone to distinguish between the virulent and the non-virulent varieties, but Neisser's stain is an efficient method of separating the Klebs-Löffler bacillus from the Hofmann varieties. Roux and Yersin were able to convert virulent diphtheria cultures into non-virulent forms by growing in broth through which a current of air was passed, but they were unable to convert the non-virulent into the virulent varieties. Pugh thinks that the work of Hewlett and Knight, who stated that they had succeeded in converting a Hofmann's bacillus into a virulent diphtheria bacillus, is not absolutely conclusive, as there seems a possibility that their original cultures were not pure. Probably the Hofmann's bacillus is never converted into the true diphtheritic variety, and certainly it never gives rise to pathological lesions.

**Bacteriology of Ophthalmia Neonatorum.**—Grœnouw [6] from the study of a hundred cases of this disease concludes that the infective organisms are as follows:—Gonococcus, 41 per cent. of cases; staphylococcus pyogenes aureus, 4 per cent.; streptococcus, 2 per cent; micrococcus luteus, 1 per cent.; B. Coli, 7 per cent. Although the gonnorrhœal infection is generally a severe one, it may sometimes be a simple catarrh, and not infrequently is associated with corneal ulcers, the prognosis in such cases being bad. The gonococcus is readily distinguished by its decolorising with Gram's stain, and may be recognised in the sac weeks after the cessation of the discharge. Forty per cent. of the cases studied were non-microbial in origin. The infections by B. Coli were probably from contact with the perineum at birth.

**Otitis Media.**—Franke [7] has studied the bacteriology of 76 cases of this disease. He concludes that there is no specific organism but it may be associated with the pneumococcus, streptococcus, staphylococcus, albus taureus, B. influenzæ, and B. of Friedlander. Infection by the bacillus diphtheriæ is not uncommon, and may be primary or secondary. It is generally associated with other organisms, such as the pseudo-diphtheria bacillus, and may remain for a long time in the discharges and thus constitute a centre of infection. Streptococcus infections are grave, but towards their termination are likely to be replaced by the staphylococcus. Chronic otitis is always polymicrobic, both pathogenic and saprophytic organisms being present. In the chronic cases, too, anærobic forms abound, and these or the bacillus pyogenes fœtidus may account for the fœtor. Although many cases are primarily tubercular, it is seldom possible to demonstrate the bacilli in the discharge.

[1] Lancet, Nov. 25, 1901. [2] Reprint from Univ. of Penn. Med. Bullet. [3] Brit. Med. Jour., Dec. 21, 1902. [4] Bullet. de Soc. Med. des Hosp. 3e serie p. 980, 1901. [5] First Ann. Rep. Hosp. Com. Metrop. Asyl. Board, 1901. [6] Archiv. f. Opthal. lii. 1. [7] Amer. Medicine, Nov. 16, 1901.

